# Compartment differences of inflammatory activity in chronic obstructive pulmonary disease

**DOI:** 10.1186/s12931-014-0104-3

**Published:** 2014-08-26

**Authors:** Jie Ji, Ida von Schéele, Jan Bergström, Bo Billing, Barbro Dahlén, Ann-Sofie Lantz, Kjell Larsson, Lena Palmberg

**Affiliations:** Lung and Airway Research, Institute of Environmental Medicine, Karolinska Institutet, Box 287, 171 77 Stockholm, Sweden; Department of dental medicine, Karolinska institutet, Stockholm, Sweden; Department of Medicine, Unit for Heart and Lung disease, Karolinska institutet, Stockholm, Sweden

**Keywords:** Chronic obstructive pulmonary disease, Tumor necrosis factor, Biomarkers, Saliva

## Abstract

**Background:**

Chronic obstructive pulmonary disease (COPD) is associated with local and systemic inflammation. The knowledge of interaction and co-variation of the inflammatory responses in different compartments is meagre.

**Method:**

Healthy controls (n = 23), smokers with (n = 28) and without (n = 29) COPD performed spirometry and dental examinations. Saliva, induced sputum, bronchoalveolar lavage (BAL) fluid and serum were collected. Inflammatory markers were assessed in all compartments using ELISA, flow cytometry and RT-PCR.

**Results:**

Negative correlations between lung function and saliva IL-8 and matrix metalloproteinase-9 (MMP-9) were found in smokers with COPD. IL-8 and MMP-9 in saliva correlated positively with periodontal disease as assessed by gingival bleeding in non-smokers.

Tumor necrosis factor-α (TNF-α) in saliva, serum and TNF-α mRNA expression on macrophages in BAL-fluid were lower in smokers than in non-smokers. There were positive correlations between soluble TNF-α receptor 1 (sTNFR1) and soluble TNF-α receptor 2 (sTNFR2) in sputum, BAL-fluid and serum in all groups. Sputum interleukin-8 (IL-8) or interleukin-6 (IL-6) was positively correlated with sTNFR1 or sTNFR2 in non-smokers and with sTNFR2 in COPD.

**Conclusion:**

Saliva which is convenient to collect and analyse, may be suitable for biomarker assessment of disease activity in COPD. An attenuated TNF-α expression was demonstrated by both protein and mRNA analyses in different compartments suggesting that TNF-α response is altered in moderate and severe COPD. Shedding of TNFR1 or TNFR2 is similarly regulated irrespective of airflow limitation.

## Introduction

Chronic obstructive pulmonary disease (COPD) is characterized by airway inflammation, chronic airflow limitation, progressive tissue destruction, extra-pulmonary manifestations and systemic inflammation. Several studies had shown the relationship between inflammatory biomarkers and exacerbations as well as systemic inflammation in COPD [1,2]. The inflammatory response in COPD is dominated by neutrophils and chemokines/cytokines such as tumor necrosis factor-α (TNF-α) and interleukin-8 (IL-8), which are of importance for neutrophils recruitment [3]. Also interleukin-6 (IL-6), a pro-inflammatory cytokine, is increased locally in the airways and systemically in COPD, especially in association with acute exacerbations [4]. TNF-α is capable of macrophage activation and stimulation of matrix metalloproteinase production [5], and the effects are mediated through interaction with tumor necrosis factor receptor 1 (TNFR1, TNF receptor 55) and tumor necrosis factor receptor 2 (TNFR2, TNF receptor 75), which are expressed on the surface of a number of cell types [6]. The TNFRs also appear in soluble forms which are generated by proteolytic cleavage of the cell surface bound TNFR in response to inflammatory mediators such as endogenous TNF-α [7]. Matrix metalloproteinase-9 (MMP-9) degrades components of the extracellular matrix which alters the balance between MMP-9 and its inhibitor [8], tissue inhibitor of metaloproteinases-1 (TIMP-1) that plays a critical role in inducing airway remodelling. Chronic destructive periodontal disease is characterized by chronic inflammation of the periodontal tissues. Smoking, which is the main risk factor for COPD, also increases the risk for periodontal disease by 5 to 20 times [9]. There are epidemiological studies suggesting a co-variation between periodontal disease and COPD [10] but a causal relationship between the two diseases has not been convincingly demonstrated.

In this cross sectional study inflammatory biomarkers, of importance in COPD were assessed in different compartments (mouth, large and small airways and blood) in smokers with and without COPD and healthy non-smokers. The aim was to find out whether or not the inflammatory processes in smokers are similarly regulated in different tissues and to what extent the presence of airway obstruction influences these outcomes.

## Materials and methods

### Subjects and study design

Twenty-three non-allergic, healthy non-smokers and 57 current smokers with a cumulative exposure of ≥5 pack-years were included. Smokers with a post-bronchodilator FEV_1_/FVC <0.7 and FEV_1_ of 40-70% of predicted value were included in the COPD group (n = 28), and smokers with a post-bronchodilator FEV_1_/FVC >0.7 and FEV_1_ > 70% of predicted value were included in the non-COPD group (n = 29) (Table 1). Spirometry was performed according to the ATS/ERS guidelines. Subjects with a history of asthma, other pulmonary disease or serious heart disease were excluded. Exacerbations during the last month prior to the study constituted an exclusion criterion.

**Table 1:** **Characteristics of the participants**

	**Healthy non-smokers**	**Smokers without COPD**	**Smokers with COPD**
Subjects n	n = 23	n = 29	n = 28
Age yrs Mean (range)	55 (41-72)	53 (38-66)	61 (48-73)**###
Gender male/female	15/8	14/15	11/17
BMI Mean (range)	25.0 (19.7-31.2)	25.1 (20.4-32.7)	23.7 (17.3-29.7)
Smoking(pack-yrs) Mean (range)	0	36 (5-120)	37 (15-60)
FEV_1%_ predicted (post- bronchodilator)	102 (97-106)	96 (91-100)	58***### (51-65)
FEV_1_/FVC (post-bronchodilator)	0.80 (0.77-0.82)	0.77 (0.75-0.79)	0.55***### (0.51-0.58)

Results are presented as mean and 95% confidence intervals or range.

Between groups comparisons were assessed by ANOVA and Fisher’s PLSD.

*BMI*: Body mass index; *FVC*: Forced Vital Capacity; *FEV*_*1*_: Forced Expiratory Volume in one second.

**, ***indicate P < 0.01 and P < 0.001, respectively, compared with healthy non-smokers.

### indicate P < 0.001 compared with smokers without COPD.

A clinical periodontal examination included assessment of periodontal pockets depth, gingival bleeding, and number of remaining teeth and occurrence of dental plaque. Periodontal tissue inflammation was assessed by gingival bleeding on probing, expressed as percentage of bleeding sites. Saliva, induced sputum and broncho-alveolar lavage (BAL) were collected on three separate days. Blood samples were drawn on the bronchoscopy day. Characterizations and periodontal status of the subjects have been described elsewhere [11].

All subjects gave their informed consent and the study was approved by the ethics committee at Karolinska Institutet, Stockholm, Sweden.

### Saliva

The subjects were not allowed to brush their teeth, eat, drink (except water) or smoke at least 90 minutes before the visit. After rinsing their mouth twice with cold water, salivation was stimulated by watching a basket of lemons. After 10 minutes collection by repeated spitting into a sterile Falcon tube, the saliva was weighed and centrifuged at 3600 rpm, at 4°C, 4 minutes. The supernatant was collected and stored in -80°C until analyses.

### Induced sputum

Sputum induction and processing were performed as previously described [12]. After inhalation of salbutamol (0.4 mg), sputum was induced by inhaling increased concentrations of saline (0.9%, 3%, 4% and 5%), by using an ultrasonic nebulizer. At each saline concentration, FEV_1_ was measured. The subjects were asked to blow their noses and rinse their mouths with water after each concentration, and then to cough deeply and to make an attempt to expectorate sputum. A sample that macroscopically appeared to be free from saliva and had a weight > 1 g was considered adequate. An equal volume of 0.1% dithiothreitol was added to the whole sputum sample and rocked for 15-20 minutes in a 37°C water bath, and then the samples were filtered and centrifuged. The supernatant was collected and stored at -80°C until analyses. The cell pellet was re-suspended using PBS and put on ice until antibody staining for flow cytometry.

Trypan blue was used to determine total cell number and cell viability. Differential cell counts were performed by counting approximately 300 cells on cytospins and stained with May-Grünwald Giemsa, and the data have been presented elsewhere [13]. Sputum samples containing more than 30% of squamous epithelial cells were excluded from the analysis.

### Broncho-alveolar lavage (BAL)

Bronchoscopy was performed as previously described [14]. After pre-medication with morphine or pethidine and scopolamine, a bronchoscopy was performed using local anesthesia with xylocaine. The bronchoscope was wedged in a middle lobe segmental bronchus and isotonic saline (5 × 50 ml) was instilled into the airway tree and gently sucked back. The lavage fluid was collected and after centrifugation, the supernatant was stored in - 80°C until analyses. The cell pellet was re-suspended, then total cell count and differential cell count were performed as done with sputum samples [13]. The cells were re-suspended in RPMI cell medium supplemented with 5% serum and then put into petri dishes at a concentration of 2 million cells/dish After 2 hours the non-adherent cells and supernatants were discarded and the adhered cells (macrophages) were prepared for mRNA analysis.

### Serum/blood

Peripheral blood was collected in supplement-free tubes and ethylene diaminetetra-acid (EDTA) vacutainer tubes (BD Bioscience, New Jersey, USA). The samples in supplement free tubes were left 60 minutes to clot and then centrifuged twice at 3000 rpm, for 10 minutes. The obtained serum was then aliquoted and stored in -70°C until analyses. The samples in EDTA vacutainer tubes were used for flow cytometry analysis.

### Flow cytometry

For TNFR1 and TNFR2 analyses of neutrophils in sputum (100 000 cells in total) and blood neutrophils and monocytes in whole blood (100 μL) were incubated with 10 μL monoclonal antibodies (anti-CD120a (TNFR1)-PE clone H398, anti-CD120b (TNFR2)-PE clone 80 M2, IOTest®) for 20 minutes. Isotype-matched antibodies were used as negative controls. After incubation, the samples were fixed and centrifuged, then stored at 2 – 8°C and analyzed within 2 hours. Analyses were performed by FACSCalibur™ and median fluorescent intensity (MFI) was determined by CELLQuest™ (BD Bioscience Pharmingen) and relative median fluorescent intensity (rMFI) was calculated.

The sTNFR1 and sTNFR2 in supernatant from sputum, BAL fluid and serum were detected using BD™ Cytometric Bead Array (CBA) flex set (BD Bioscience Pharmingen). Analyses were performed by FACSCalibur™ and the concentration of sTNFR1 and sTNFR2 were determined by FCAP Array™ Software (BD Bioscience Pharmingen). The range of the standard curve was 0-10000 pg/ml for sTNFR1 and 0-2500 pg/ml for sTNFR2.

### mRNA preparation and real-time PCR

Preparation of mRNA from alveolar macrophage was performed in eight subjects from each group. Total mRNA was isolated by PureLink™ Micro-to-Midi Total RNA Purification System (Initrogen). DNase I, amplification grade was used to remove the genomic DNA (Initrogen). First-strand cDNA was synthesized from 0.5 μg of total mRNA, using QuantiTect® Reverse Transcription Kit (Qiagen, Hilden, Germany). The ABI Power SYBR Green Master mix (Applied Biosystems) was used to perform the RT-PCR, also the primers were purchased from Invitrogen. The widely used cDNA of glyceraldehydes-3-phosphate dehydrogenase (GAPDH) was adopted as an internal control gene. 1 μl cDNA was used in each 25 μl PCR reaction volume to identify the products of interest.

Data were analyzed using 7500 Software v.2.0.1, the results were then calculated and expressed as 2^-ΔCt^.

### ELISA

Measurement of IL-6 and IL-8 in saliva, sputum and BAL-fluid was performed using an in- house ELISA [15]. The lower detection limit of the IL-6 assay was 3 pg/ml in all compartments. The limit of IL-8 assay of saliva and sputum was 50 pg/ml and for BAL was 12.5 pg/ml.

MMP-9 and TIMP-1 in supernatant from saliva, sputum, BAL-fluid and serum were measured using purchased DouSet ELISA MMP-9 Kit and DouSet ELISA TIMP-1 Kit (R&D SYSTEMS®). The measurements of TNF-α in supernatant from saliva, sputum and serum were performed by purchased HS quantikine ELISA Kit (R&D SYSTEMS®). The analyses of MMP-9, TIMP-1 and TNF-α were performed according to the manufacturer. For all the duplicated samples, an intra-assay variation <10% (for TNF-α, <20%) was accepted.

### High-sensitivity CRP test

A high-sensitivity CRP (hs-CRP) test was used to measure serum level of C-reactive protein (CRP) with laser nephelometry.

### Statistics

Depending on distribution of the data results are presented as means (95% confidence intervals) or medians (25 - 75 percentiles). Between groups comparisons were assessed by ANOVA followed by Fisher’s protected least significant difference (PLSD), or by Kruskal- Wallis test with the Mann-Whitney U test as a post hoc test when appropriate, and by means of Spearman’s rank correlation. A p-value <0.05 was considered significant. All data were analyzed using StatView version 5.0.1 (SAS Institute Inc., Cary NC).

## Results

### Clinical characteristics and dental signs

Characteristics of the subjects are shown in Table 1. Smoking habits were approximately 35 pack-years in both smoker groups. An impaired periodontal status was found in both smokers with and without COPD with no major difference between the two groups as previously described [11].

### Saliva

**Figure 1:**
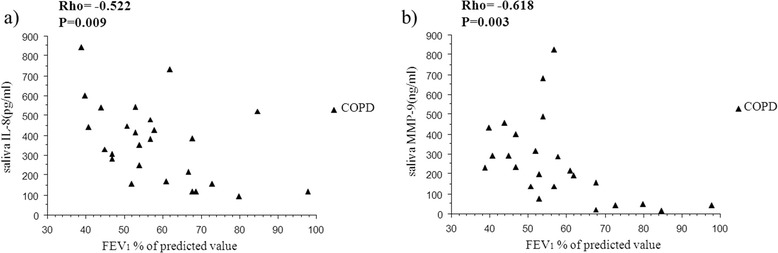
**Relationship between lung function index and salivary biomarkers.** Relationship between FEV_1_ (percent of predicted value) and IL-8 **(a)** and MMP-9 **(b)** in saliva in smokers with COPD. Rho indicates coefficient of correlation according to Spearman.

**Figure 2:**
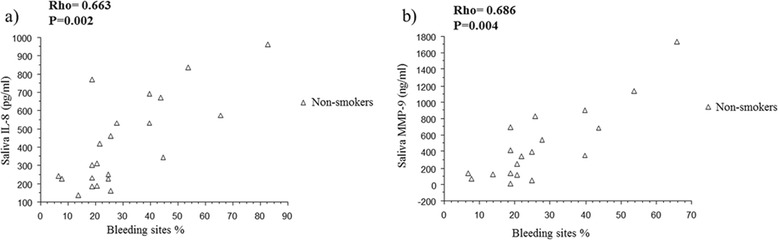
**Relationship between dental statues and salivary biomarkers.** Relationship between gingival bleeding index and IL-8 **(a)** and MMP-9 **(b)** in saliva in healthy non-smokers. Rho indicates coefficient of correlation according to Spearman.

The level of salivary TNF-α was significantly lower in the two smoker groups than in non- smokers (P < 0.001) whereas IL-8, MMP-9 and TIMP-1 in saliva did not differ between the groups (Table 2). There was a negative correlation between lung function and the salivary levels of IL-8 and MMP-9 in the COPD group (Figure 1). Gingival bleeding correlated positively with IL-8 and MMP-9 levels in saliva of non-smokers (Figure 2).

**Table 2:** **Inflammatory mediators in saliva**

	**Healthy non-smoker**	**Smokers without COPD**	**Smokers with COPD**	
IL-8 (pg/ml)	307 (225-558)	262 (208-407)	360 (165-474)	P = 0.512
MMP-9 (ng/ml)	338 (105-679)	170 (62-465)	217 (101-351)	P = 0.437
TIMP-1 (ng/ml)	449 (225-814)	354 (221-535)	360 (241-469)	P = 0.556
MMP-9/TIMP-1	0.54 (0.29-1.16)	0.37 (0.23-1.10)	0.38 (0.27-0.96)	P = 0.789
TNF-α (pg/ml)	2.04 (1.19-3.82)	0.529*** (<0.5-1.25)	0.704*** (<0.5-1.23)	**P < 0.001**

Results are presented as median and 25^th^-75^th^ percentiles.

P-values indicate comparisons between groups (Kruskal-Wallis test), bold data indicate significance.

***indicate P < 0.001 compared with healthy non-smokers (Mann-Whitney U test).

### Sputum

Both groups of smokers had higher levels of IL-6 in sputum than had non-smokers (P < 0.001), and sputum IL-8 was higher in COPD patients than in the non-smokers (P = 0.006; Table 3). TNF-α level in sputum was under detection limit in almost all cases. Sputum neutrophil TNFRs expression and sputum sTNFRs did not differ between the groups. The levels of sputum IL-6 and IL-8 were positively correlated with sputum sTNFR1 and sTNFR2 in healthy controls, and with sputum sTNFR2 in COPD patients (Figure 3).

**Table 3:** **Inflammatory mediators in respiratory tracts**

		**Healthy non-smoker**	**Smokers without COPD**	**Smokers with COPD**	**P-value**
Sputum	IL-6 (pg/ml)	33.4 (11.5-62.6)	223*** (78.9-641)	486*** (262-1031)	**P < 0.001**
	IL-8 (pg/ml)	628 (430-1351)	1100 (595-1710)	2941**# (980-4912)	**P = 0.010**
	MMP-9 (ng/ml)	21.6 (13.3-109)	57.3 (30.2-106)	97.5 (17.4-408)	P = 0.091
	TIMP-1 (ng/ml)	117.5 (28.7-257)	96.6 (46.4-242)	166 (69.4-346)	P = 0.423
	MMP-9/TIMP-1	0.35 (0.14-1.36)	0.78 (0.19-1.72)	0.70 (0.22-1.80)	P = 0.636
	TNF-α (pg/ml)	1.17 (<1.00-4.88)	<1.00 (<1.00)	<1.00 (<1.00-8.05)	P = 0.123
	sTNFR1 (pg/ml)	589 (59-996)	205 (36-536)	80 (16-560)	P = 0.207
	sTNFR2 (pg/ml)	140 (65-469)	196 (118-344)	204 (43-545)	P = 0.996
	TNFR1 on neutrophils (rMFI)	1.94 (1.46-2.48)	2.70 (2.36-2.94)	1.76 (1.44-2.12)	P = 0.076
	TNFR2 on neutrophils (rMFI)	1.58 (1.37-1.67)	1.63 (1.35-1.93)	1.34 (1.17-1.49)	P = 0.236
BAL	IL-8 (pg/ml)	<12.5 (<12.5-15.8)	<12.5 (<12.5-33.0)	20.3 (<12.5-31.8)	P = 0.459
	MMP-9 (ng/ml)	0.69 (0.44-1.45)	0.97 (0.58-1.27)	1.38 (0.93-3.87)	P = 0.112
	TIMP-1 (ng/ml)	1.85 (1.46-3.71)	3.92** (2.71-9.34)	3.46** (2.18-7.85)	**P = 0.010**
	MMP-9/TIMP-1	0.51 (0.18-0.77)	0.25 (0.11-0.62)	0.42 (0.11-0.74)	P = 0.654
	sTNFR1 (pg/ml)	82.2 (75.4-118)	161** (102-203)	92.9 # (73.7-133)	**P = 0.009**
	sTNFR2 (pg/ml)	36.0 (24.8-50.2)	77.7** (54.6-110)	69.5* (34.6-88.5)	**P = 0.003**
	Macrophages TNF-α (2 ^-∆Ct^)	0.05 (0.04-0.12)	0.02* (0.02-0.03)	0.01* (0.01-0.05)	**P = 0.027**
	Macrophages TNFR1 (2 ^−^ ∆Ct_)_	0.008 (0.005-0.01)	0.006 (0.005-0.016)	0.004*# (0.004-0.005)	**P = 0.037**
	Macrophages TNFR2 (2 ^−^ ∆Ct_)_	0.003 (0.001-0.003)	0.003 (0.003-0.004)	0.002 (0.001-0.002)	P = 0.055

Results are presented as median and 25^th^-75^th^ percentiles.

P-values indicate comparisons between groups (Kruskal-Wallis test), bold data indicate significance.

In BAL fluid IL-6 was below detection limit (<3 pg/ml) with a few exceptions and TNF-α was below detection limit (<0.5 pg/ml) in all samples.

*, **, ***indicate P < 0.05 P < 0.01 and P < 0.001, respectively, compared with healthy non- smokers (Mann-Whitney U test).

# indicate P < 0.05 compared with smokers without COPD (Mann-Whitney U test).

**Figure 3:**
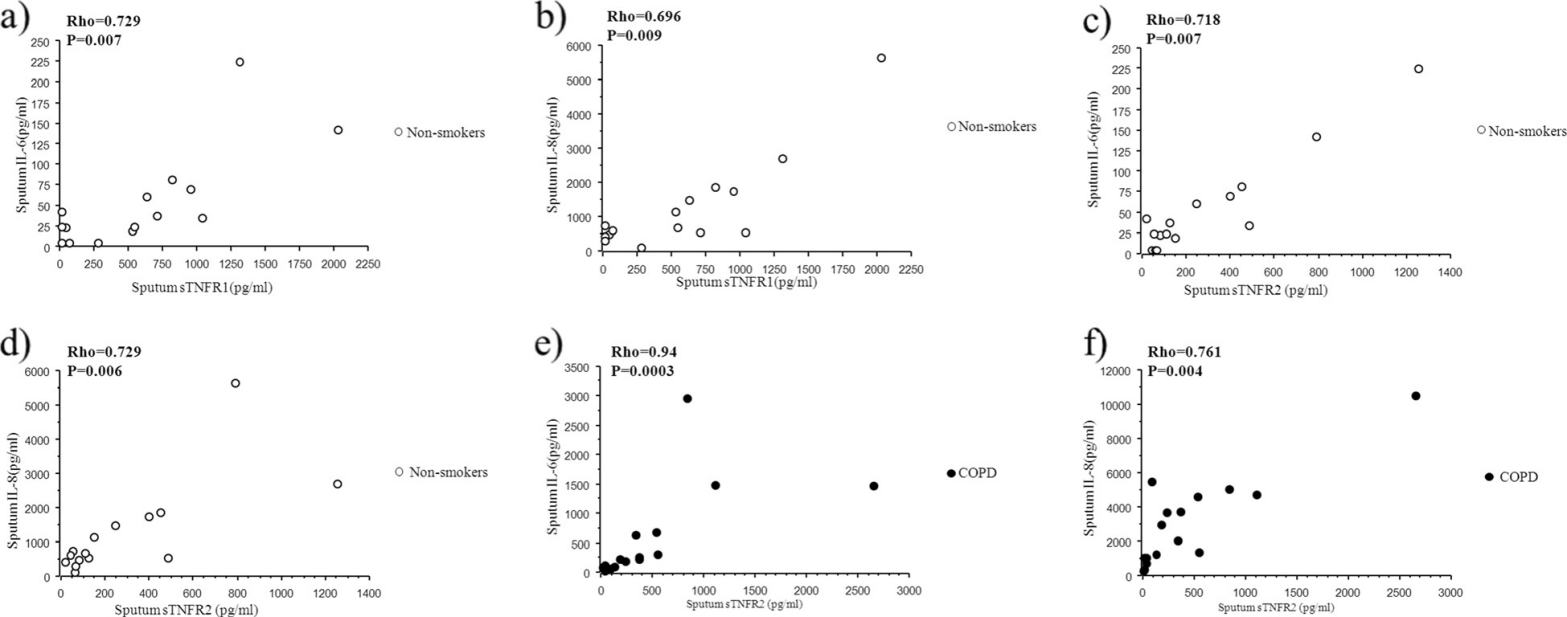
**Relationship between sputum soluble TNFRs and sputum biomarkers.** Relationship between sputum concentration of soluble TNF- α receptors and IL-6 and **(a, c)** and IL-8 **(b, d)** in healthy non-smokers. Relationship between sputum concentration of sTNFR2 and IL-6 and **(e)** and IL-8 **(f)** in smokers with COPD. Rho indicates coefficient of correlation according to Spearman.

### Bronchoalveolar lavage (BAL)

In BAL-fluid, IL-8 was under detection limit in most cases and MMP-9 did not differ between the groups (P = 0.112; Table 3). The levels of TIMP-1 were higher in the two smoker groups than in non-smokers (P = 0.01; Table 3). The macrophage mRNA TNF-α was lower in both smoker groups than in the non-smokers (P = 0.027) while its receptor (TNFR1) was higher in COPD group than the other two groups (P = 0.037; Table 3). sTNFR1 levels in BAL fluid were higher in smokers without COPD than in the other two groups, and sTNFR2 levels were increased in both smoker groups compared with non-smokers (Table 3).

### Serum/blood

TNF-α in serum was lower in the COPD group than in other two groups (P = 0.049; Table 4).

**Table 4:** **Inflammatory mediators in serum/blood**

		**Healthy non-smoker**	**Smokers without COPD**	**Smokers with COPD**	
Serum	MMP-9 (ng/ml)	430 (251-577)	490 (382-801)	757** (557-1000)	**P = 0.006**
	TIMP-1 (ng/ml)	282 (221-367)	358 (266-442)	338 (298-544)	P = 0.104
	MMP-9/TIMP-1	1.21 (0.92-1.93)	1.31 (0.96-2.29)	2.13 (1.57-2.81)	P = 0.116
	CRP (mg/l)	0.75 (0.40-1.20)	1.80** (1.00-2.70)	2.45** (0.73-4.30)	**P = 0.004**
	TNF-α (pg/ml)	1.39 (0.72-1.93)	1.22 (1.08-1.53)	0.64*# (<0.5-1.30)	**P = 0.049**
	sTNFR1 (pg/ml)	1353 (817-1570)	1322 (1166-1855)	1302 (943-1829)	P = 0.848
	sTNFR2 (pg/ml)	3499 (2806-3708)	3561 (3097-3919)	3750 (2975-4261)	P = 0.826
Blood	TNFR1 on monocytes (rMFI)	11.8 (10.5-15.0)	12.5 (9.9-13.7)	13.2 (11.3-15.7)	P = 0.600
	TNFR2 on monocytes (rMFI)	28.2 (21.3-47.8)	31.1 (25.0-40.9)	44.0 (28.9-52.2)	P = 0.342
	TNFR1 on neutrophils (rMFI)	6.95 (6.24-9.45)	6.74 (5.42-7.70)	6.69 (5.70-7.92)	P = 0.509
	TNFR2 on neutrophils (rMFI)	11.8 (8.79-14.6)	8.28 (7.30-9.97)	7.82 (6.58-10.6)	P = 0.117

Results are presented as median and 25^th^-75^th^ percentiles.

P-values indicate comparisons between groups (Kruskal-Wallis test), bold data indicate significance.

IL-6 and IL-8 were below detection limit (<3 pg/ml, <12.5 pg/ml) in serum in almost all subjects.

*, **indicate P < 0.05 and P < 0.01, respectively, compared with healthy non-smokers (Mann-Whitney U-test).

# indicate P < 0.05 compared with smokers without COPD (Mann-Whitney U-test).

The levels of CRP in serum were lower in non-smokers than in the two groups of smokers with no difference between the latter two (P = 0.004; Table 4). Serum MMP-9-level was higher in COPD than in healthy controls (P = 0.006; Table 4).

### TNFR1 and TNFR2

**Figure 4:**
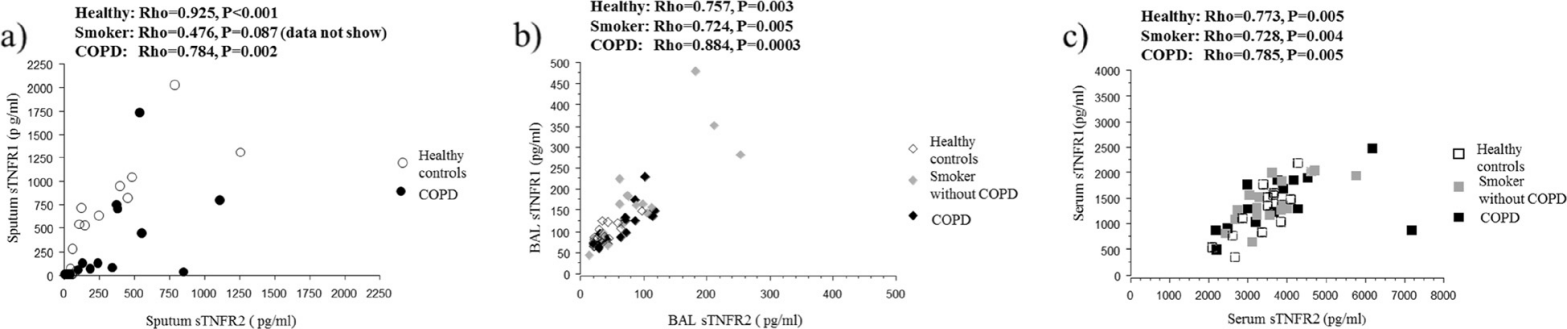
**Relationship between soluble TNFR1 and TNFR2.** Relationship between soluble TNFR1 and TNFR2 in sputum **(a)**, BAL fluid **(b)** and serum **(c)**. Rho indicates coefficient of correlation according to Spearman.

There was a significant positive correlation between sTNFR1 and sTNFR2 both in BAL fluid and serum in all three groups (Figure 4). In sputum, there was a significant correlation between sTNFR1 and sTNFR2 in healthy controls and in smokers with COPD (Figure 4) but not in smokers without COPD (Rho = 0.476; P = 0.09).

Pooled data from all three groups revealed a positive correlation between TNFR1 and TNFR2 expression on circulating neutrophils (Rho = 0.843; P = 0.0001; data not shown).

## Discussion

In the present study it was shown that smokers have an ongoing inflammation in the central airways (sputum), peripheral airways (BAL fluid), and systemically (blood) and that this inflammatory response is rather associated with smoking than with the presence or absence of chronic airflow limitation.

Although the levels of IL-8 and MMP-9 did not differ between the groups there was a significant negative relationship between saliva levels of IL-8 and MMP-9 and lung function in COPD. The findings may indicate that the inflammatory markers in saliva may be related to disease severity in COPD. A similar relationship has previously been shown between biomarkers in serum and lung function [16]. However whether or not that saliva analyses may be useful for analyses of inflammatory markers in COPD has to be further explored. Intriguingly, we found a very strong correlation between IL-8 and MMP-9 in saliva and periodontal inflammation assessed by gingival bleeding in healthy non-smokers but not in the two groups of smokers. This finding indicates that these markers of inflammation in saliva are associated with periodontal inflammation under normal conditions and that this association is masked in smokers when inflammatory activity is triggered by a potent pro- inflammatory stimulus such as tobacco smoke.

The levels of TNF-α in saliva and serum as well as TNF-α mRNA expression in macrophages in BAL fluid were lower in smokers with COPD than in non-smokers. The attenuated TNF-α response was thus demonstrated in different compartments (mouth, serum, alveolar macrophages) by the use of different methods (protein and mRNA expression) clearly indicating a generally diminished TNF production and secretion. It has previously been shown that pro-inflammatory cytokine, e.g. TNF-α, responses to different stimuli are attenuated in macrophages and monocytes from smokers compared with non-smokers [17–19]. In a study by Pinto-Plata et al. [20], there was a positive relationship between the blood levels of TNF-α and the severity of the disease in patients with COPD and the patients with moderate disease (GOLD stage II) had the lowest blood levels of TNF-α [21]. Di Francia et al. [22] demonstrated unaltered TNF-α in patients with severe COPD who did not lose weight while COPD patients with unintentional weight loss had high TNF-α serum levels. Our COPD patients had normal BMI and were in stage II and III with a FEV_1_ > 40% of predicted value implying that, patients with low BMI and the most severe disease were not included. The clinical effect of TNF-α inhibitors has been shown to be poor in mild and moderate.

COPD [23,24] whereas an improved physical performance of TNF-α inhibition has been indicated in COPD patients with severe disease and cachexia [25]. It could thus be speculated that the TNF-α response in COPD is bimodal with an attenuated response in moderate disease and an enhanced response in more severely ill patients; in particular those who also experience weight loss. In addition, our COPD patients were in a stable phase with no recent exacerbations. According to Sapey et al. [26] TNF-α is quiescent in stable COPD and becomes biologically active during exacerbations which is in agreement with the low TNF-α activity found in our stable COPD patients.

Interestingly, sTNFR1 and sTNFR2 were strongly correlated in sputum, BAL fluid and serum as was TNF-receptor expression on blood neutrophils. We therefore conclude, although the production of TNFRs is assumed to use different signaling pathways , the regulation and shedding of TNFRs occur in parallel on a cellular level, as assessed by TNFR expression on neutrophils, locally in BAL fluid and sputum and systemically in serum.

The levels of sTNFRs in BAL fluid were higher in both groups of smokers than in non- smokers. It has previously been reported that high levels of sTNFRs may regulate TNF-α formation [27] and compete with membrane bound TNFRs [28]. Therefore, it seems likely that the high levels of sTNFRs in the smokers may inhibit the production of membrane bound TNFRs. This is in agreement with our finding of lower mRNA expression of TNFRs on BAL macrophages from both groups of smokers compared with non-smokers. In addition, as macrophages are a major producer of TNF-α [6], it could be speculated that macrophages respond to inflammation by regulating the levels of TNFRs along two different lines, enhancement of TNFR shedding and reduced production of membrane bound TNFRs.

The increased levels of the MMP-9 in extracellular matrix is of importance for remodeling processes in COPD, and its expression is considered to be regulated by specific inhibitors, such as TIMP-1 [29]. We found elevated levels of TIMP-1 in BAL fluid from both groups of smokers compared with healthy non-smokers and increased levels of MMP-9 in serum in the COPD group. Elevated levels of MMP-9 and TIMP-1 have been observed in serum [30], sputum [31] and BAL fluid in COPD [32,33]. However, there are contradicting results indicating decreased plasma levels of MMP-9 and TIMP-1 in COPD [33]. These inconsistent results might be due to the fact that MMP levels may vary over time in COPD [33]. Differences in the severity of the disease and smoking habits in the study population may also explain differences between studies.

In conclusion, we demonstrated that saliva, which is easy to collect, might be suitable for studies of biomarkers in smokers with and without COPD. Also, our study provides comprehensive information about different inflammatory biomarkers in different compartments and showed associations of different inflammatory markers both locally and systemically in smokers with and without COPD. An attenuated local and systemic TNF-α response as assessed both by mRNA and protein analyses was demonstrated in moderate COPD. Furthermore, a close relationship between TNF-α receptor expression and other inflammatory markers as well as between two different soluble TNF-α receptors was shown.
